# Attritional Extensor Digiti Minimi Tendon Rupture Associated With a Distal Ulna Fracture

**DOI:** 10.7759/cureus.44893

**Published:** 2023-09-08

**Authors:** Cordero L McCall, Emmanuel Dean, Lilah Fones, Pedro Beredjiklian

**Affiliations:** 1 Orthopaedic Surgery, Medical College of Wisconsin, Milwaukee, USA; 2 Orthopaedic Surgery, Rothman Orthopaedic Institute, Philadelphia, USA; 3 Orthopaedic Surgery, Morehouse School of Medicine, Atlanta, USA; 4 Orthopaedic Surgery, Thomas Jefferson University Hospital, Philadelphia, USA; 5 Division of Hand Surgery, Rothman Orthopaedic Institute, Philadelphia, USA

**Keywords:** arthritis, tendon transfer, extensor digiti minimi, extensor tendon rupture, distal ulna fracture

## Abstract

Attritional extensor tendon ruptures are common in the setting of arthritis but, to our knowledge, have never previously been reported in the setting of a distal ulna fracture. This case report describes a 56-year-old male patient who sustained a left-hand dog bite resulting in crush injuries to the thumb and ring finger and a minimally displaced distal ulna fracture. The patient initially underwent appropriate surgical intervention for the thumb and finger crush injuries and non-operative management of the distal ulna fracture with splint immobilization. He experienced an extensor digiti minimi tendon (EDM) rupture two and a half weeks post-operatively. Radiographs demonstrated interval distal ulna fracture displacement with a prominent dorsal spike and absence of arthritis. He subsequently underwent distal ulna open reduction internal fixation and an extensor indicis proprius (EIP) to EDM tendon transfer. This case demonstrates a novel complication following non-operative management of a distal ulna fracture in which the prominent dorsal distal ulna resulted in direct irritation to the extensor tendon and subsequent attritional extensor tendon rupture. This potential complication should be considered in determining appropriate treatment for distal ulna fractures.

## Introduction

Attritional ruptures of the extensor tendons at the wrist most often occur in the setting of degenerative or inflammatory changes in the distal radioulnar joint (DRUJ) [1,2]. Ruptures result from infiltration of inflammatory synovium into the tendons or due to prominence of the distal ulna or an ulnar osteophyte at the joint [3]. Patients often present with the sudden inability to extend their fingers, with the small finger affected most often [4,5]. We present a case of a musician who developed a rupture of the extensor digiti minimi (EDM) tendon in the distal forearm secondary to a distal ulna fracture. There was no evidence of preexisting arthritis. The patient was treated with open reduction and internal fixation of the fracture, and extensor indicis proprius (EIP) to EDM tendon transfer to maintain independent small finger extension to be able to continue playing his instrument, the importance of which has been demonstrated in a prior case report by Watkins C et al. [6]. We are not aware of any other report in the literature describing an extensor tendon rupture at the wrist in the setting of a distal ulna fracture.

## Case presentation

**Figure 1:**
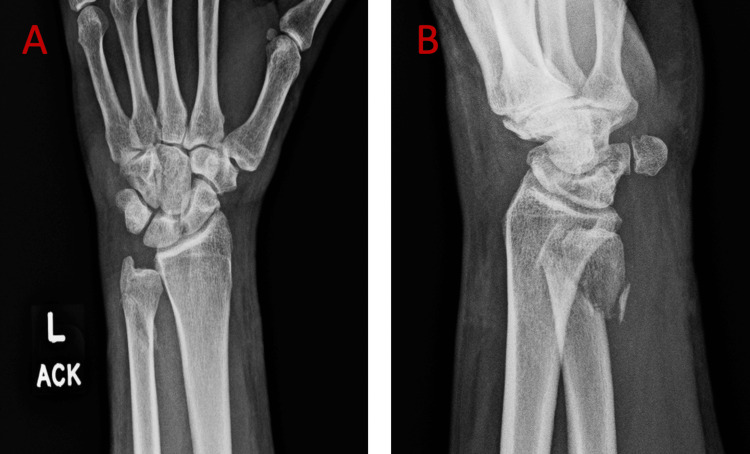
Anterior-posterior (A) and lateral (B) X-ray views of the left wrist on initial presentation revealing a minimally displaced fracture of the distal ulna.

A 56-year-old man presented to the office having sustained a dog bite to the left non-dominant hand for follow-up post-acute injury. On presentation, crush injuries to the thumb and ring finger and a minimally displaced, closed fracture of the distal ulna were identified (Figure 1). Irrigation with 3 liters of antibiotic solution and debridement of crush injuries to the thumb and ring fingers were performed. At the same time, the ulna was treated conservatively with volar wrist splint immobilization immediately post-op. Twelve days post-operatively, his sutures were removed, the exam demonstrated normal finger range of motion, and he was placed into a custom fabricated forearm-based thumb spica splint. 

**Figure 2:**
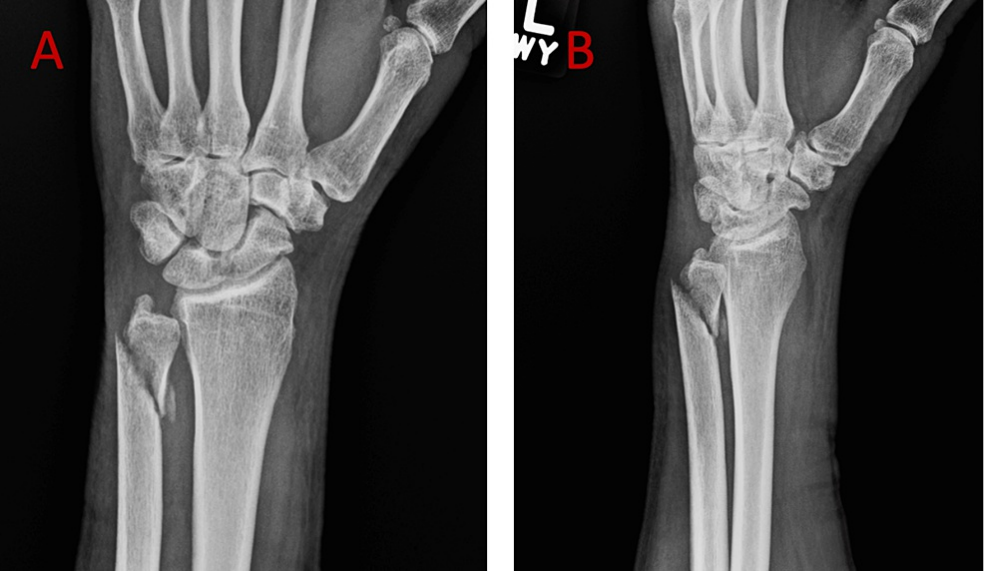
Anterior-posterior (A) and lateral (B) X-ray views of the left wrist 2-3 weeks post-injury showing interim displacement of the distal ulna fracture with a dorsal proximal bony spike.

The early post-surgical course was uneventful until two and a half weeks post-op, when the patient noted a sudden onset of the inability to extend the small finger, prompting him to return to the office for repeat evaluation. On examination, the patient was found to have a small finger weakness and extensor lag at the metacarpophalangeal (MP) joint. A sagittal band exam revealed no evidence of extensor tendon subluxation at the MP joint. Tenodesis testing revealed no passive extension of the small finger with wrist flexion. A neurologic exam was intact, with specific attention to the radial and posterior interosseous nerve. X-rays obtained in the office revealed interim displacement of the distal ulna fracture with a dorsal proximal bony spike (Figure 2).

**Figure 3:**
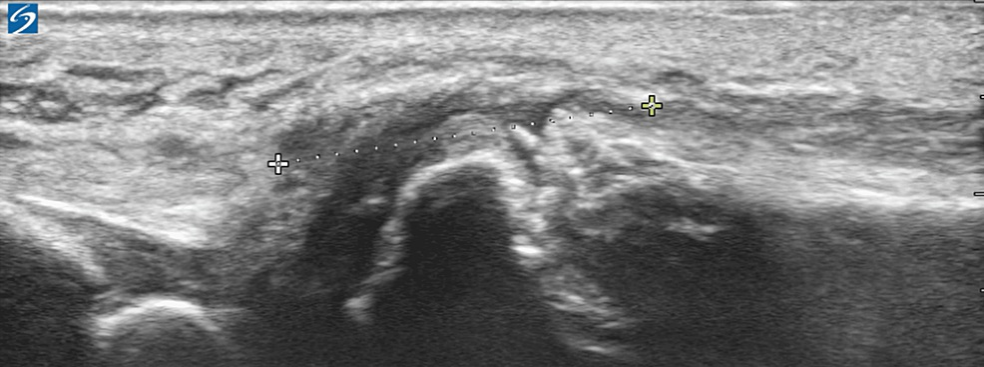
Ultrasound image demonstrating a complete EDM rupture with a 2cm gap and a partial EDC tear. EDM: Extensor digiti minimi tendon' EDC: Extensor digitorum communis.

An ultrasound evaluation of the wrist confirmed a complete rupture of the EDM tendon with a two-centimeter gap and a partial tear of the extensor digitorum communis to the small finger (Figure 3).

**Figure 4:**
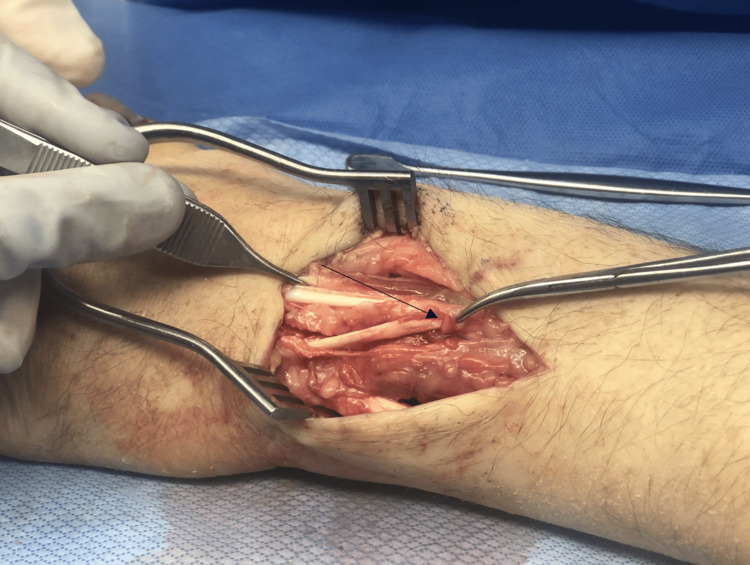
Intraoperative image revealing the extensor digiti minimi tendon rupture in the area of the distal ulna fracture.

The patient was brought back to the operating room, where he underwent an open reduction and internal fixation of the distal ulna fracture with headless compression screws. The EDM tendon complete rupture with surrounding tenosynovitis was confirmed intraoperatively (Figure 4), and an extensor indicis proprius (EIP) to EDM transfer was performed using a Pulver-Taft weave under appropriate tensioning to allow independent small finger extension [6].

**Figure 5:**
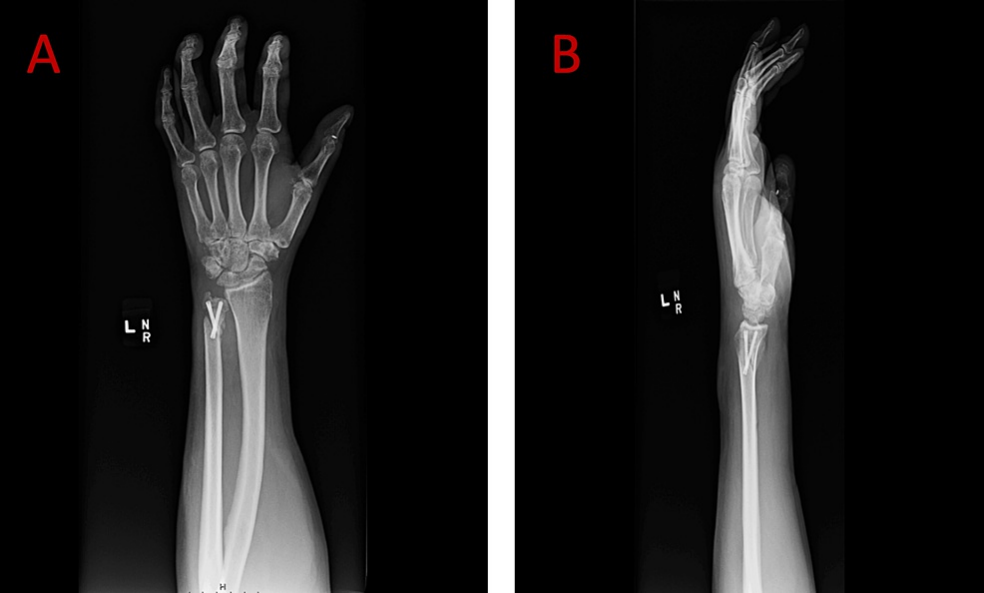
Anterior-posterior (A) and lateral (B) X-ray views of the left wrist at six weeks post distal ulna open reduction and internal fixation using headless compression screws.

At six weeks post-operatively, the exam improved small finger extension strength and range of motion. Radiographs demonstrated interval fracture healing and appropriate alignment (Figure 5).

## Discussion

This case report presents an attritional extensor tendon rupture two weeks following a non-operatively treated distal ulna fracture. Extensor tendon injury can broadly be defined as traumatic or attritional injury. Attritional ruptures are commonly associated with rheumatoid arthritis, occurring in approximately 4% of rheumatoid patients undergoing wrist operations in the context of osteoarthritis and after distal radius fracture [7-11]. Yamazaki H et al. reported tendon ruptures in 37 patients with osteoarthritis, noting an increased risk of tendon rupture with associated scallop sign (sclerotic border of the ulnar aspect of distal radius), dorsal inclination of the sigmoid notch, and radial shift of the ulnar head relative to the radius. Case reports have also reported attritional extensor tendon ruptures in distal ulnar resection for DRUJ arthritis [12,13], scaphoid nonunion advanced collapse (SNAC) wrist [14], and Kienbock's disease [15]. Traumatic, non-arthritic tendon injuries most often result from incidents involving sharp objects or saws, typically affecting the dominant hand [16]. However, case reports have also documented closed traumatic extensor tendon ruptures at the myotendinous junction [17]. To our knowledge, an extensor tendon rupture following a non-operatively treated distal ulna fracture has not been previously reported in the literature.

The etiology of the EDM tendon rupture in this case is unclear, but we hypothesize that it occurred due to direct irritation from the prominent dorsal ulna fracture. Similar mechanisms of attritional extensor tendon rupture have been observed in the context of osteoarthritis and post distal radius excision. Both Yamazaki H et al. and Carr AJ et al. have reported extensor tendon ruptures where prominent dorsal distal radioulnar joint osteophytes abutted the 4th and 5th dorsal wrist compartments [8,9]. A similar mechanism was described by Fletcher C et al., where they demonstrated an extensor tendon rupture following distal radius excision. This rupture was attributed to a prominent dorsal distal ulna causing direct irritation to the extensor tendon, leading to an attritional rupture [12]. Similarly, in this case, we propose that the attritional EDM rupture was likely caused by the EDM tendon abutting the distal ulna fracture, resulting in direct tendon irritation and subsequent rupture.

Distal ulna fractures, including ulnar styloid and ulnar metadiaphyseal fractures, have an incidence of approximately 74/100,000, with the majority being associated with radius fractures [18]. Internal fixation of the distal ulna can present challenges given the small size of the distal fragments. Currently, there is no consensus on the treatment for distal ulna fractures [19]. One study, which examined 766 patients treated for distal ulnar shaft fractures, found that when ulnar styloid fractures were excluded, only 30% of the distal one-third ulna fractures underwent open reduction internal fixation (ORIF). Of these, only 10 of the 48 surgically treated distal ulna fractures were isolated ulna fractures [18]. Research on isolated ulna fractures is limited; however, previous studies have shown no significant advantage between nonoperative and operative treatments in terms of bone healing and complications, with no post-operative extensor tendon ruptures reported in either group [20]. Of note, a common extensor tendon can produce a complete extension of the 5th digit without an EDM tendon, indicating that this presentation could often be present but overlooked [9]. Given our patient's EDM rupture two weeks post-operation, this could suggest a potential role for ulna ORIF during the initial surgery. This case report has several limitations. First, our patient presented with a dog bite, which could have caused an unrecognized EDM partial tear that made it more susceptible to attritional rupture. However, the absence of bite marks on the dorsal side of the wrist makes this less probable. Additionally, the lack of long-term follow-up data and functional outcome assessments limits our understanding of the patient's outcome. Moreover, our findings are based on a single middle-aged patient with no prior symptoms or indications of osteoarthritis, rheumatoid, or other connective tissue diseases, which restricts its general applicability. Further studies are required to understand the mechanism of extensor tendon rupture and ascertain the frequency of this complication. Despite these limitations, the study provides valuable insights into a novel complication with nonoperative management of distal ulna fractures that should be considered when weighing the risks and benefits of operative and nonoperative management of distal ulna fractures.

## Conclusions

Isolated distal ulna fractures are an uncommon injury, and no consensus exists on the indications for operative management. We present the case of a 56-year-old male who experienced an EDM tendon rupture two and a half weeks after non-surgical treatment of a distal ulna fracture. When weighing the pros and cons of isolated distal ulna fracture ORIF, the presence of dorsal bony prominence at the fracture site may favor operative intervention, given the potential for direct irritation to the extensor tendons and subsequent attritional rupture. Further studies are essential to determine the extent of distal ulna fracture displacement that becomes risky in terms of extensor tendon rupture.
